# Threshold conditions for curbing COVID-19 with a dynamic zero-case policy derived from 101 outbreaks in China

**DOI:** 10.1186/s12889-023-16009-8

**Published:** 2023-06-06

**Authors:** Sanyi Tang, Xia Wang, Biao Tang, Sha He, Dingding Yan, Chenxi Huang, Yiming Shao, Yanni Xiao, Robert A. Cheke

**Affiliations:** 1 School of Mathematics and Statistics, Shaanxi Normal University, Xi’an, 710119 P.R. China; 2 Center for Intersection of Mathematics and Life Sciences, Xi’an Jiaotong University, Xi’an, 710049 P.R. China; 3 Beijing Changping Laboratory, Beijing, 102299 P.R. China; 4 Natural Resources Institute, University of Greenwich at Medway, Central Avenue, Chatham Maritime, Kent, ME4 4TB UK; 5 Department of Infectious Disease Epidemiology, School of Public Health, Imperial College London, St Mary’s Campus, Norfolk Place, London, W2 1PG UK

**Keywords:** COVID-19, Non-pharmaceutical interventions, Mathematical model, Epidemic waves, Mitigation, China

## Abstract

By 31 May 2022, original/Alpha, Delta and Omicron strains induced 101 outbreaks of COVID-19 in mainland China. Most outbreaks were cleared by combining non-pharmaceutical interventions (NPIs) with vaccines, but continuous virus variations challenged the dynamic zero-case policy (DZCP), posing questions of what are the prerequisites and threshold levels for success? And what are the independent effects of vaccination in each outbreak? Using a modified classic infectious disease dynamic model and an iterative relationship for new infections per day, the effectiveness of vaccines and NPIs was deduced, from which the independent effectiveness of vaccines was derived. There was a negative correlation between vaccination coverage rates and virus transmission. For the Delta strain, a 61.8% increase in the vaccination rate (VR) reduced the control reproduction number (CRN) by about 27%. For the Omicron strain, a 20.43% increase in VR, including booster shots, reduced the CRN by 42.16%. The implementation speed of NPIs against the original/Alpha strain was faster than the virus’s transmission speed, and vaccines significantly accelerated the DZCP against the Delta strain. The CRN (${R}_{c1}$) during the exponential growth phase and the peak time and intensity of NPIs were key factors affecting a comprehensive theoretical threshold condition for DZCP success, illustrated by contour diagrams for the CRN under different conditions. The DZCP maintained the ${R}_{c1}$ of 101 outbreaks below the safe threshold level, but the strength of NPIs was close to saturation especially for Omicron, and there was little room for improvement. Only by curbing the rise in the early stage and shortening the exponential growth period could clearing be achieved quickly. Strengthening China's vaccine immune barrier can improve China's ability to prevent and control epidemics and provide greater scope for the selection and adjustment of NPIs. Otherwise, there will be rapid rises in infection rates and an extremely high peak and huge pressure on the healthcare system, and a potential increase in excess mortality.

**Supplementary Information:**

The online version contains supplementary material available at 10.1186/s12889-023-16009-8.

## Introduction

**Fig. 1:**
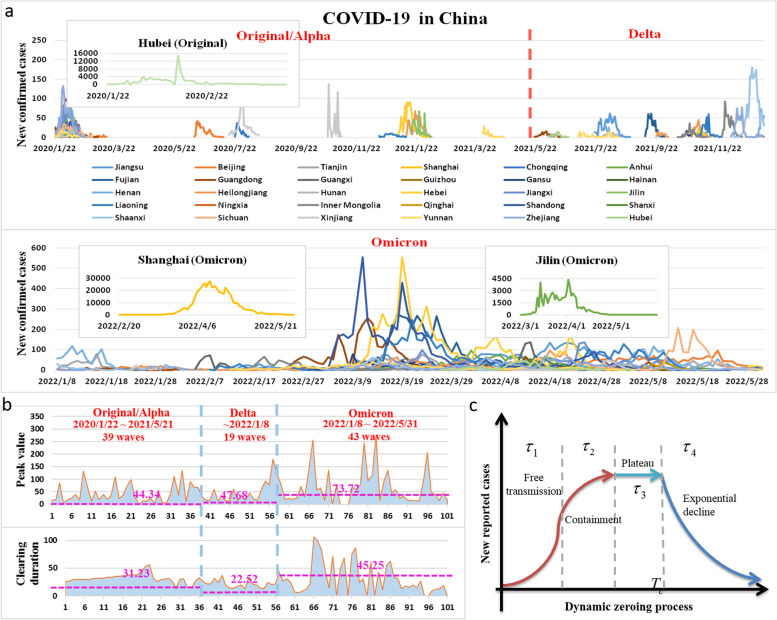
101 epidemic waves in mainland China and data analyses. **a** Time series of 101 epidemic waves (from 1 Jan 2020 to 31 May 2022) in mainland China caused by the original/Alpha, Delta and Omicron strains, with three large-scale outbreaks including those in Hubei, Shanghai and Jilin individually marked; **b** The mean peak values and durations of clearing times of 101 epidemic waves for different virus strains. When calculating the average value of the peak value, we excluded the data for five Provinces and cities exceeding 500 due to the peak values of these five provinces being significantly higher than those of other provinces, identified as outliers by Boxplot, namely 14,840 in Hubei (original/Alpha, 13 February 2020), 27,605 in Shanghai (Omicron, 13 April 2022), 555 in Shandong (Omicron, 11 March 2022), 4427 in Jilin (Omicron, 2 April 2022) and 555 in Hebei (Omicron, 19 March 2022). **c** Four stages of the epidemics including the free rising period with regular epidemic NPIs (${\tau }_{1}$), containment exponential growth period (${\tau }_{2}$), plateau period (${\tau }_{3}$) and exponential decline period (${\tau }_{4}$). The duration of clearing times (${T}_{c}={\tau }_{1}+{\tau }_{2}+{\tau }_{3}+{\tau }_{4}$) can be calculated from the real time series and the theoretical formula given in the Methods (Extended Data Online content)

During past three years of the COVID-19 pandemic, prevention and control strategies in China have been changed from containment in the early stage to the dynamic zero-case policy (DZCP), and then to almost complete re-opening recently [1–3]. Before November 2022, China's epidemic prevention strategy was mainly to implement a rapid DZCP through locking down cities, large-scale and almost complete coverage of tests with nucleic acid screening, close contact tracking and isolation, and improving vaccine coverage. By combining the above strong non-pharmaceutical interventions (NPIs) with vaccines, before 31 May 2022, 101 outbreaks of spatially concentrated COVID-19 infections caused by the original/Alpha strain and Delta and Omicron mutant strains in China were quickly and dynamically cleared (Fig. 1a) [4]. Due to the sensitive monitoring system, each outbreak was detected in its early initial stage followed by early isolation and treatment measures. Consequently, outbreak areas were locked down or partially locked down quickly, and then continuous nucleic acid screening was carried out for all personnel until the goal of zero clearance was achieved, resulting in nearly 100% close contact tracking, isolation and nucleic acid screening rates within a short period, otherwise the DZCP strategy could not have been realized so quickly for each outbreak.

However, due to the characteristics of the Omicron virus such as its high infectivity and low pathogenicity, China has adjusted its prevention and control measures after implementing the DZCP for nearly three years. Notably, “20 measures” were announced on 11 November 2022 which included reduced isolation periods, the stopping of mass testing except when a source of infection was known, relaxing travel restrictions and associated testing, boosting healthcare resources and stockpiling medicines, followed by a further “10 new measures” on 7 December 2022 [5, 6]. These mainly allowed people with mild or asymptomatic infections to be quarantined at home and reduced the frequency of nucleic acid testing, according to a statement issued by the State Council's Joint Prevention and Control Mechanism. Also, since 8 January 2023 Covid-19 has been down-graded from a Category A to a Category B disease http://www.nhc.gov.cn/xcs/zhengcwj/202301/bdc1ff75feb94934ae1dade176d30936.shtml. Since then, Omicron infections have spread rapidly in major cities including Beijing, Shanghai, Chongqing and Chengdu where the Omicron epidemics have been putting severe pressure on the healthcare system since late December 2022 [7, 8].

Compared with each outbreak wave before and after the policy adjustment, we found that before the adjustment, the goal of DZCP could be achieved in a relatively short time, the peak time of each wave was very short and the peak numbers of infections and final sizes of outbreaks were very small. After the adjustment, not only is it impossible to achieve the goal of the DZCP, but also the epidemic situation in various regions reaches a peak quickly with a huge peak value. Therefore, we can infer that there is a threshold for the intensity of NPIs to achieve the goal of dynamic zeroing. The key problem is how to determine this threshold and the extent that the independent effect of the vaccine has played in the process of dynamic zeroing for each outbreak. Answering the above two questions can provide important decision-making guidance for the prevention and control of COVID-19 or the adjustment of prevention and control policies for new emerging infectious diseases.

## Methods

### Data collection and statistical analyses

We obtained data on laboratory-confirmed COVID-19 cases in China from the National Health Commission of the People’s Republic of China (http://www.nhc.gov.cn/xcs/xxgzbd/gzbd_index.shtml), as shown in Fig. 1a [4]. As of 31 May 2022, there had been 101 outbreaks of the COVID-19 epidemic in China, including 39 outbreaks of the original/Alpha strain, 19 outbreaks of the Delta strain and 43 outbreaks of the Omicron strain. If the number of newly reported cases was zero for three consecutive days, we considered that the epidemic had been dynamically cleared, and small-scale epidemics with peak values of less than or equal to 10 were not counted in the 101 epidemic waves (each wave had a peak with a peak value). Moreover, in the 101 outbreaks few or no COVID-related deaths were reported, except for during the first outbreak of the Wuhan epidemic induced by the original Alpha strain and during the Shanghai epidemic induced by the Omicron strain. Therefore, we did not collect the numbers of daily reported deaths for the 101 outbreaks in this study. Powerful and high-frequency nucleic acid detection in China made it impossible to accurately distinguish between symptomatic and asymptomatic patients because infected people were detected in time at an early stage, and consequently there was almost no under-reporting of cases.

The outbreak of the Delta strain started on 21 May 2021 in Guangdong, and the outbreak of the Omicron strain started on 8 January 2022 in Tianjin. We collected the numbers of daily reported cases for these 101 outbreaks from the National Health Commission. Using the maximum number of newly reported cases per day in each outbreak, the mean peak value during the epidemic period of each virus strain can be calculated (Fig. 1, Supplementary materials: Extended Data Fig. 1). In addition, no matter what kind of virus strain induced each wave, the dynamic zero-case policy (DZCP) [1–3] could be achieved within about 40 days, and consequently we denote the duration of each outbreak as the clearing duration. The mean value of the clearing duration can be calculated directly by using the duration of each wave. As a result, the average peaks were 44.34, 47.68 and 73.72 cases and the average durations were 31.23, 22.52 and 45.25 days for outbreaks caused by the Alpha, Delta and Omicron strains, respectively, (Fig. 1b). It is noted that the data used to calculate the mean peak value do not include the five outbreaks with peaks greater than 500, namely 14,840 (Alpha, 13 February 2020) in Hubei, 27,605 (Omicron, 13 April 2022) in Shanghai, 555 (Omicron, 11 March 2022) in Shandong, 4427 (Omicron, 2 April 2022) in Jilin and 555 (Omicron, 19 March 2022) in Hebei. The peak values of these five provinces were significantly higher than those of other provinces and were identified as outliers by Boxplot. Besides, at the set research termination time, there were still 7 outbreaks of the Omicron strain that had not ended. What we are faced with here is right-censored, for which the complete duration time has been cut off at the deadline. Therefore, we chose the Kaplan–Meier approach to estimate a survivor function and further calculated the mean survival time, which refers to the mean duration time here. The result of the survival analysis was 45.25, and its 95% confidence interval was (34.77, 55.73) (Fig. 1). The 7 outbreaks have now ended so we also calculated the mean duration times for them as 44.30, based on the real data, which verified the reliability of the survival analysis results.

In addition to the numbers of daily reported cases, we also collected vaccination data, including the number of daily vaccination injections in some provinces of China from 15 December 2020 to 24 January 2022 (Supplementary materials: Extended Data Tables 1 and 2). Thus, the vaccination ratio is defined as the ratio of the total number of injections to the total population (Supplementary materials: Extended Data Table 1). Here, when calculating the corresponding vaccination ratio for the epidemic caused by the Delta variant, we used the data on the total number of injections at the start of the epidemic. However, due to the lack of later vaccination data (the vaccination data are as of 24 January 2022), the vaccination ratio of the epidemic induced by the Omicron variant was calculated using the vaccination data on 24 January 2022 (Supplementary materials: Extended Data Table 2).

#### Dynamic model

With strict follow-up quarantine, isolation and treatment measures related to China's DZCP, we employed a general Susceptible-Infected-Recovered (SIR)-type epidemiological model with contact tracing developed by Keeling & Rohani [9], because all of the reported cases were treated in isolation, and the powerful NPIs effectively avoided the impact of the incubation period of each virus strain on the detected cases. Considering the close tracking and isolation measures that were implemented in China, we divided the population into the following groups of people: susceptible ($S$), infected ($I$), quarantined (both susceptible and infected) (${I}_{q}$) and recovered/confirmed ($R$) [4]. Let $N$ be a constant to denote the total population, the transmission probability be $\beta$, contact rate be a constant $c$, quarantined rate be $q$ and the confirmation rate of the quarantined infected people be ${\updelta }_{q}$. $1/\gamma$ represents the transmission period with various NPIs being effective. Here, it is assumed that the quarantined susceptible population ${(I}_{q})$ will not return to the susceptible population before the dynamic zeroing of the epidemic due to strong NPIs and short epidemic duration. The model is as follows:

1 $$\left\{\begin{array}{l}{S}^{^{\prime}}=-\frac{\left(\beta c+cq\left(1-\beta \right)\right)}{N}SI,\\ {I}^{^{\prime}}=\frac{\beta c(1-q)}{N}SI-\gamma I, \\ {S}_{q}^{^{\prime}}=\frac{(1-\beta )cq}{N}SI, \\ {I}_{q}^{^{\prime}}=\frac{\beta cq}{N}SI-{\delta }_{q}{I}_{q}, \\ {R}^{^{\prime}}={\delta }_{q}{I}_{q}+\gamma I.\end{array}\right.$$

Note that dynamic zeroing for each wave could be achieved in about 40 days, and that the closely tracked and quarantined susceptible population generally needs to be quarantined for two weeks in a centralized manner and one week at home, so that the quarantined susceptible population will not become susceptible again at the end of a wave. We emphasize here that a SEIR model rather than a SIR model based on the transmission mechanism of COVID-19 could be employed. However, due to the powerful high-frequency nucleic acid testing, most infected people will not have experienced a complete process from infection to incubation period, and then to asymptomatic or symptomatic, i.e. every patient may be found at every stage after infection, and thus a SIR model (1) has been used here. Because of this, the epidemic data only include the number of newly reported confirmed cases. Of course, it would be possible to develop mathematical models incorporating more practical factors, including age structure, and conduct more in-depth research [10].

As mentioned in the data analyses, our study excluded several outbreaks with more than 500 newly reported cases in a single day, and all outbreaks can be dynamically cleared in about 40 days. Therefore, compared with the total population size of each province or municipality (at least tens of millions and at most hundreds of millions), the S/N ratio is basically 1 in the process of each outbreak under China's powerful NPI strategy. For example, as of 31 May 2022, the largest proportion of the total number of infected cases to the total population of each province was Shanghai (2.52%), followed by Jilin (0.31%), and Hubei (0.12%), all of which were excluded from this study for the reasons explained above. The prevalence in the regions used in this study are shown in Extended Data Fig. 1. Therefore, it is reasonable to assume that $\frac{S}{N}\approx 1$ always held during each outbreak in mainland China before the relaxation of control measures, which results in:

2 $$\left\{\begin{array}{c}{I}^{^{\prime}}=\beta c\left(1-q\right)I-\gamma I=\gamma {(R}_{c}-1)I,\\ {{I}_{q}}^{^{\prime}}=\beta cqI-{\delta }_{q}{I}_{q}=\frac{\upgamma q}{1-q}{R}_{\mathrm{c}}I- {\delta }_{q}{I}_{q}.\end{array}\right.$$

Let ${\beta }_{1}=\left(\beta c+cq\left(1-\beta \right)\right)$, ${\beta }_{2}=\beta c\left(1-q\right)$, ${\beta }_{3}=\beta cq,$ the control reproduction number (CRN) ${R}_{\mathrm{c}}=\frac{{\beta }_{2}}{\gamma }$, the basic reproduction number ${R}_{0}=\frac{\beta c}{\gamma }$, then ${\beta }_{3}=\beta cq=\frac{\upgamma q}{1-q}{R}_{\mathrm{c}}$. Note that the ${R}_{\mathrm{c}}$ is determined by the transmission probability $\beta$ which is directly affected by the effectiveness of vaccines, contact rate $c$, quarantined rate $q$ and the transmission period $1/\gamma$ which are directly affected by the strength of NPIs. Solving the equation for $I$ in (2) yields:

3 $$I\left(\mathrm{t}\right)={I}_{0}\mathrm{exp}[\upgamma {(R}_{\mathrm{c}}-1)\mathrm{t}].$$

Substituting Eq. (3) into the second equation of (2), we have

$${{I}_{q}}^{\mathrm{^{\prime}}}={\beta }_{3}{I}_{0}\mathrm{exp}[\upgamma {(R}_{\mathrm{c}}-1)\mathrm{t}]-{\delta }_{q}{I}_{q}.$$

Taking the initial value as 0 and solving the above differential equation gives:

$${I}_{q}\left(t\right)=\left[\frac{{\beta }_{3}{I}_{0}\mathrm{exp}[{(R}_{\mathrm{c}}\upgamma +{\delta }_{q}-\upgamma )\mathrm{t}]-{\beta }_{3}{I}_{0}}{{R}_{\mathrm{c}}\upgamma +{\delta }_{q}-\upgamma }\right]\mathrm{exp}\left(-{\delta }_{q}t\right).$$

It follows from Eq. (2) that the number of newly reported cases in the controlled area is $\frac{\upgamma q}{1-q}{R}_{\mathrm{c}}I$ and the number of newly reported cases in the uncontrolled area is $\upgamma {R}_{c}I$. Note that due to very extensive and frequent nucleic acid testing, the total number of newly reported cases is exactly the total number of new infections. Moreover, the total number of daily reported cases at time t can be calculated as follows:

$$\mathrm{New}\left(t\right)=\frac{\upgamma q}{1-q}{R}_{\mathrm{c}}I+\upgamma {R}_{\mathrm{c}}I=\frac{\upgamma }{1-q}{R}_{\mathrm{c}}I=\frac{\upgamma {R}_{\mathrm{c}}{I}_{0}}{1-q}\mathrm{exp}[\upgamma {(R}_{\mathrm{c}}-1)\mathrm{t}].$$

Furthermore, we have the following iteration formula for the number of daily reported cases:

4 $$\mathrm{New}\left(t+1\right)=f\left(\mathrm{New}\left(t\right)\right)=\mathrm{New}(t)\mathrm{exp}[\gamma {(R}_{c}-1)].$$

The iteration relation shown in (4) contains only two parameters, namely, the transmission period $\frac{1}{\gamma }$ and the CRN ${R}_{c}$, which has involved the combined effect of NPIs and vaccine efficacy. This relationship is equivalent to the well-known one obtained for SIR models [11]. Further, we can consider $\upgamma {(R}_{\mathrm{c}}-1)$ as a whole in the iterative formula (4) with the exponential growth rate as the only parameter, which can provide useful information on characterizing the transmissibility.

#### Estimation of the epidemic duration

Based on the epidemic data and times for implementing NPIs for each outbreak, as shown in Fig. 1c, the dynamic zeroing process of each outbreak may experience four distinct phases: (1) a free rising period with regular epidemic prevention and control (${\tau }_{1}$); (2) a containment rising period (${\tau }_{2}$); (3) a plateau period (${\tau }_{3}$) and (4) an exponentially declining period (${\tau }_{4}$). Note that the exponential growth period can be calculated as ${\tau }_{1}+{\tau }_{2}$, and with strong NPIs some of the outbreaks considered in this work have no plateau period and/or no free rising period [1–3]. We used a linear regression model to describe the relationship between the logarithm of the number of daily reported cases ($y$) and the time $t$ in the exponential growth period and the exponential declining period, as follows:

5 $$y={r}_{i}t+{b}_{i}, i=\mathrm{1,2}$$

where ${r}_{1} (\mathrm{or} {r}_{2})$ is the exponential growth (or decline) rate, ${b}_{1} ({b}_{2})$ is the intercept term. As is well known, the first confirmed case is often reported later than the onset of an epidemic. If the exponential growth rate of the number of daily reported cases at the beginning of the epidemic is assumed to be ${r}_{1}$, the onset time of the epidemic can be calculated by $y=0$, namely ${t}_{0}=$-$b/{r}_{1}$. Suppose the reporting time of the first confirmed case is ${\mathrm{t}}_{1}$= 1, then the free rising period can be determined to be

6 $${\tau }_{1}={t}_{1}-{t}_{0}= \frac{b}{{r}_{1}}+1.$$

This simple formula can deal well with the problem of reporting delay, and the free rising period for 6 epidemics induced by Omicron and for 4 epidemics induced by Delta strains based on Eq. (6) as shown in Extended Data Table 4.

#### The estimation of changing points

The four distinct phases of each outbreak are determined by three key switching points, and here an analytical method to estimate these three switching points was employed [12, 13], to further identify the time nodes of each stage, determined on the basis of the actual data and adjustment of the prevention and control strategies. We used Bayes’s method to capture the transition time from the free rising stage to the containment stage, and the transition time from the containment stage to the plateau stage. Since the linear regression model (5) can well describe the change of the logarithmic value of the number of new cases, we introduced change points into the model and then estimated the change points based on the data. A posterior distribution expression of a single change point for a general switching linear model is available [11, 12]. By giving the prior information about the parameters, the times when the three phases switch can be obtained by sampling using the posterior distribution. The estimated switching points of the first three phases by sampling using the posterior distribution are given in the first three columns of Extended Data Table 4.

#### Calculation of the CRN

With the strengthening of NPI measures, especially the improvement of detection, the infection period of undiagnosed patients was shortened. Therefore, for an infected individual, his/her transmission period is assumed to be ${1/\gamma }_{1}$ (or ${1/\gamma }_{2}$) during the exponential growth (or declining) period of the outbreak, and the corresponding CRN is ${R}_{c1}$ (or ${R}_{c2}$). According to the iterative formula for the number of daily reported cases (4), we can obtain the relationship between the exponential growth (or decline) rate and the CRN as follows:

$${r}_{i}={\gamma }_{i}\left({R}_{ci}-1\right), i=\mathrm{1,2}.$$

Thus, the CRN can be calculated from

7 $${R}_{ci}=\frac{{r}_{i}}{{\gamma }_{i}}+1, i=\mathrm{1,2}$$

According to (7), the value of the CRN depends on the value of ${r}_{i}$ and ${\gamma }_{i}$. Here, considering that the value of the transmission period may affect the value of the CRN, we calculated ${R}_{c1}$ when the transmission period is 5 and 6 days, and ${R}_{c2}$ when the infection period is 1 and 2 days (Supplementary materials: Extended Data Table 3). In addition, the epidemic situation in Shanghai experienced three adjustments to its exponential growth rate (0.39, 0.24, 0.18) before reaching the plateau period. The values of the CRN calculated by using the three exponential growth rates are, respectively, 3.34, 2.44 and 2.08 (or 2.95, 2.20, 1.90) when ${\gamma }_{1}=1/6$(${or \gamma }_{1}=1/5$), and the values given in Extended Data Tables 1 and 3 are the values for the first exponential growth stage.

#### Estimation of epidemic duration

Considering the four (or three) stages of each wave of the 101 epidemics, the role of all NPI measures is embodied in gradually reducing the exponential growth rate of the number of daily reported cases, and finally to less than 0, that is, from exponential growth to exponential decline. In other words, the CRN is reduced from ${R}_{c1}$ to ${R}_{c2}$ due to the continuous strengthening of NPIs, so we can define the relatively strengthened NPIs in this process as ${\mathrm{S}}_{\mathrm{c}}$, i.e.

$${\mathrm{S}}_{\mathrm{c}}=\frac{{R}_{c1}-{R}_{c2}}{{R}_{c1}}.$$

Thus, the threshold condition for disease control is ${R}_{c2}<1$, namely

8 $${\mathrm{S}}_{\mathrm{c}}>\frac{{R}_{c1}-1}{{R}_{c1}}.$$

Let ${T}_{c}$ be the clearing duration. Obviously, ${T}_{c}={\tau }_{1}+{\tau }_{2}+{\tau }_{3}+{\tau }_{4}$. Given the value of the NPI strength ${\mathrm{S}}_{\mathrm{c}}$, we can get the influence of the exponential growth time ${\tau }_{1}+{\tau }_{2}$ and the CRN ${R}_{c1}$ on the clearing duration ${T}_{c}$. Specifically, assume that one infected individual is introduced into the population at time 0, then the number of newly reported cases at time ${\tau }_{1}+{\tau }_{2}$ gives:

$$\mathrm{New}\left({\tau }_{1}+{\tau }_{2}\right)=\mathrm{exp}[{\gamma }_{1}{(R}_{c1}-1)({\tau }_{1}+{\tau }_{2})].$$

Because ${\tau }_{3}$ is the duration of the plateau phase, the numbers of daily reported cases remain unchanged. Thus, we have

$$\mathrm{New}\left({\tau }_{1}+{\tau }_{2}+{\tau }_{3}\right)=\mathrm{exp}[{\gamma }_{1}{(R}_{c1}-1)({\tau }_{1}+{\tau }_{2})]$$

It follows from $\mathrm{New}\left({T}_{c}\right)=\mathrm{ New}\left({\tau }_{1}+{\tau }_{2}+{\tau }_{3}\right)\mathrm{exp}[{\gamma }_{2}{(R}_{c2}-1)({T}_{c}-({\tau }_{1}+{\tau }_{2}+{\tau }_{3}))]= 1$  that

9 $${T}_{c}=\left({\tau }_{1}+{\tau }_{2}\right)\left(1+\frac{{{\gamma }_{1}(R}_{c1}-1)}{{\gamma }_{2}{(1-R}_{c2})}\right)+{\tau }_{3}\triangleq g\left({R}_{c1}, {\tau }_{1}+{\tau }_{2}, {S}_{c}\right).$$

Therefore, if condition (8) holds, the epidemic can then be eliminated, consequently the goal of dynamic zeroing can be achieved. Thus, given the CRN $\left({R}_{c1}\right),$ we can estimate the clearing duration ${T}_{c}$ according to Eq. (9) under different exponential growth durations ${\tau }_{1}+{\tau }_{2}$ and the relatively strengthened NPIs ${\mathrm{S}}_{\mathrm{c}}$. Here, the duration of the plateau phase is usually unpredictable, so we assume that ${\tau }_{3}=0$ when calculating the epidemic duration theoretically.

#### Estimation of the relatively strengthened NPIs (${\mathrm{S}}_{\mathrm{c}}$)

Define ${c}_{0}$ to be the average number of contacts before the outbreak which was 14 (or 20) [14]. It is assumed that the number of contacts has been reduced by 1/3, 1/2 or 2/3 in the exponential growth stage (${{\varvec{c}}}_{1}$), that is, the average numbers of contacts were 9.3, 7, 4.7 (or 13.3, 10, 6.7). It is assumed that the infection period in the exponential growth stage is 6 days, and that in the exponential decline stage it is 2 days (effects of these two parameters on the CRN are shown in Extended Data Table 3). Then the infection period is reduced from 6 to 2, and the CRN is reduced to 1/3 of its original value ${(R}_{c1}$). If the contact number is further reduced by another 1/3, 1/2 or 2/3, then the CRN is constrained to be 2/9, 1/6 or 1/9 of its original value ${(R}_{c1}$). Thus, increasing the detection strength and controlling social distancing reduced the CRN by 78%, 83% or 89%. The average contacts under different circumstances are shown in Extended Data Table 6. Obviously, the average contacts in bold are smaller than the average number of people in a Chinese family, which is impossible. In addition, the infection period of 2 days already limits the effect of NPI measures. Therefore, a reasonable setting is that the maximum mean of the relatively strengthened NPI ${\mathrm{S}}_{\mathrm{c}}$ is 0.89. In Extended Data Table 7, the sensitivity analyses of the CRNs and the relatively strengthened NPIs ${\mathrm{S}}_{\mathrm{c}}$ in the exponential decline stage for different ${1/\gamma }_{2}$ $({\mathrm h\mathrm e\mathrm r\mathrm e\;\mathrm w\mathrm e\;\mathrm f\mathrm i\mathrm x\mathrm e\mathrm d\;\mathrm\gamma}_1=\;1/7)$ are listed.

## Results

**Fig. 2:**
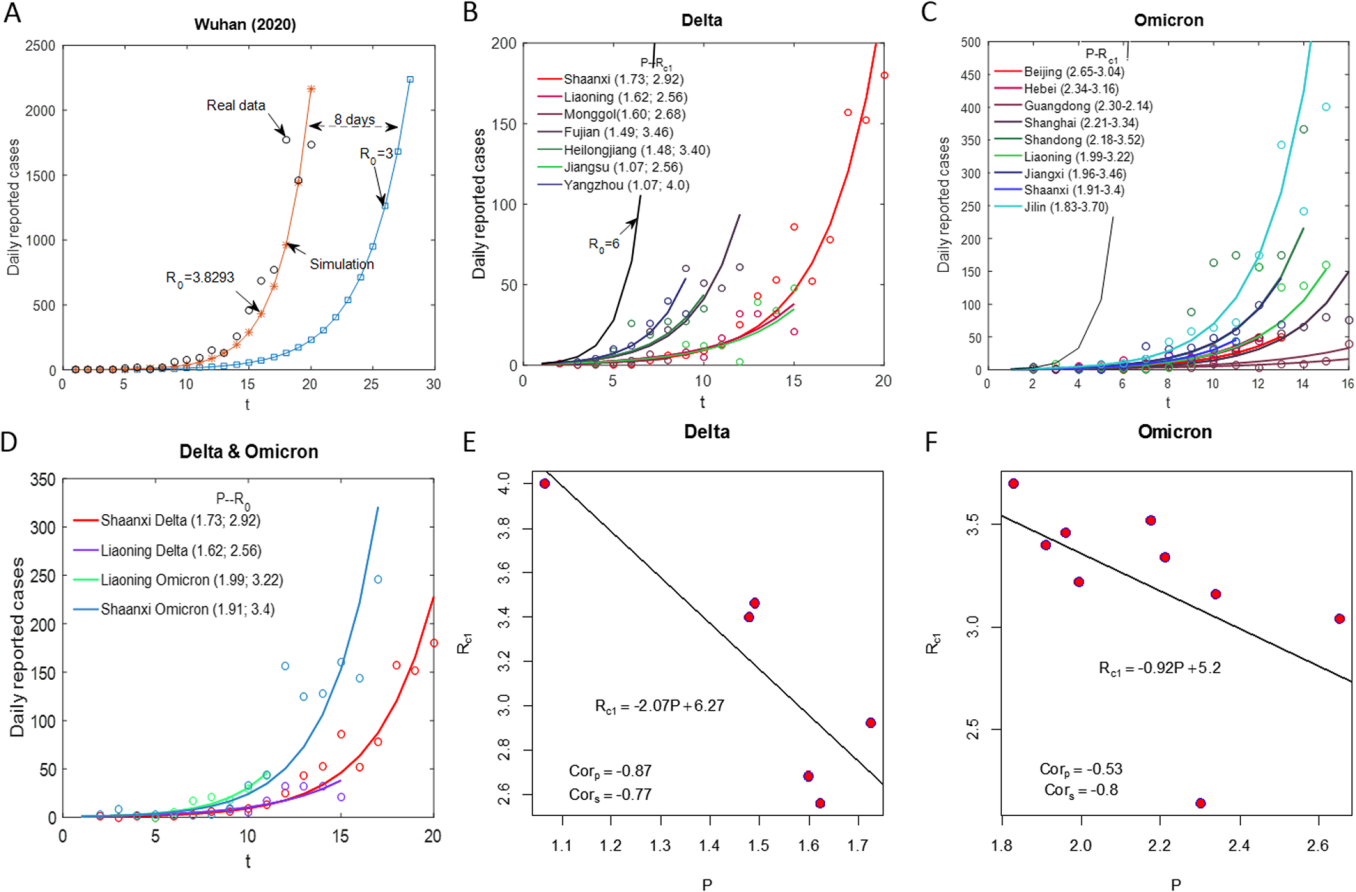
Analysis of synergistic and independent effects of vaccine and NPIs. **a** Comparison between the exponential growth curve obtained when the ${R}_{0}$ of the original variant is 3 (blue curve) and the exponential growth curve during the free rising period of the epidemic (red curve) in Wuhan, in 2020. **b** Early relevant information on 6 outbreaks caused by the Delta mutant in Shaanxi, Liaoning and other places in China in 2021 (for comparative purposes, data for Yangzhou City in Jiangsu Province are also provided), including vaccination rate (proportion of total doses to total population) and the control reproduction number (CRN) ${R}_{c1}$. **c** The early relevant information on 9 outbreaks caused by the Omicron mutant in Hebei, Guangdong, Tianjin and other places in China in 2022, including the vaccination rate (by 28 January 2022) and the values of the CRN ${R}_{c1}$. **d** Comparison of two outbreaks in Shaanxi and Liaoning caused by Delta and Omicron mutants in 2021 and 2022 indicates that even under stronger NPIs and higher vaccine coverage rate, the Omicron strain is more infectious in China. **e** Correlation analysis and linear regression between vaccination rate and ${R}_{c1}$ for 6 outbreaks caused by the Delta mutant. ${Cor}_{p}$ and ${Cor}_{s}$ represent the Pearson and Spearman correlation ceoficents, respectively. **f** Correlation analysis and linear regression between vaccination rate and ${R}_{c1}$ for 9 outbreaks caused by the Omicron mutant. ${Cor}_{p}$ and ${Cor}_{s}$ represent the Pearson and Spearman correlation ceoficents, respectively

The classic infectious disease dynamic model was used after modification and an iterative relationship for new infections per day was derived [9], and then the effectiveness of vaccines and NPIs was deduced. Initially, we simulated the new infections with ${R}_{0}=3$ to calculate a theoretical simulation curve (TSC, blue in Fig. 2a). Curve fitting (red in Fig. 2a) for the real data (from 10 to 29 January 2020) resulted in ${R}_{0}=3.82$ for the original strain in Wuhan city, which is clearly higher than the blue curve [1–3]. It is known that the very limited ability to detect the new coronavirus in early 2020 led to incomplete and late diagnoses of the initial cases which contributed to a serious underestimation of the transmission risk of the original strain involved in the Wuhan epidemic [15–17].

In 2021, the numbers of newly reported cases caused by the Delta mutant in Shaanxi (mainly in Xi’an city), Liaoning, Inner Mongolia, Fujian, Heilongjiang and Jiangsu Provinces (Yanghzou city in Jiangsu) were lower than their respective TSCs when ${R}_{0}=6$ (Fig. 2b) [16]. This shows that the combination of vaccinations and NPIs effectively curbed the exponential growth of the Delta mutant epidemic for the above six regions with ${R}_{c1}$= 2.92, 2.56, 2.68, 3.46, 3.40, 2.56 (4.0 for Yangzhou city) respectively, and the vaccination ratios corresponding to each outbreak time (number of doses / total population) were 1.73, 1.62, 1.60, 1.49, 1.48 and 1.07, respectively (Supplementary materials: Extended Data Table 1). A comparison between the epidemic caused by the Delta strain in Xi’an city with that in Yangzhou city in 2021 indicates that the 61.8% increase in the vaccination coverage rate in Shaanxi induced the 27% reduction in the CRN, conditional upon a similar intensity of NPIs, that is, the independent protective effect of the vaccine is obvious given the consistent intensity of NPIs [18, 19].

Since the Omicron mutant entered China in 2022, it has caused small-scale epidemics in 7 provinces and cities including Tianjin, Hebei and Guangdong, as well as large-scale epidemics in Jilin and Shanghai. The number of early infections increased exponentially, but at rates that were all far below the TSC for the Omicron strain with R_0_ = 8, as their ${R}_{c1}$ values were in the 2.14–3.70 range (Fig. 2c, Supplementary materials: Extended Data Table 2) [20]. Similarly, comparing the epidemics caused by the Omicron strain in Guangdong Province with those in Jilin Province in 2022 indicates that the 42.16% increase in vaccination coverage rate in Guangdong induced the 34.05% reduction in CRN, which, together with vaccination coverage rates, implies that the booster shot for COVID-19 is more effective to protect against the Omicron variant than against the Alpha or Delta variants. There is a strong negative correlation between the vaccination level and the CRN of COVID-19 [21–24]. The higher the vaccination level, the lower the CRN of the epidemic (Fig. 2d-f).

**Fig. 3:**
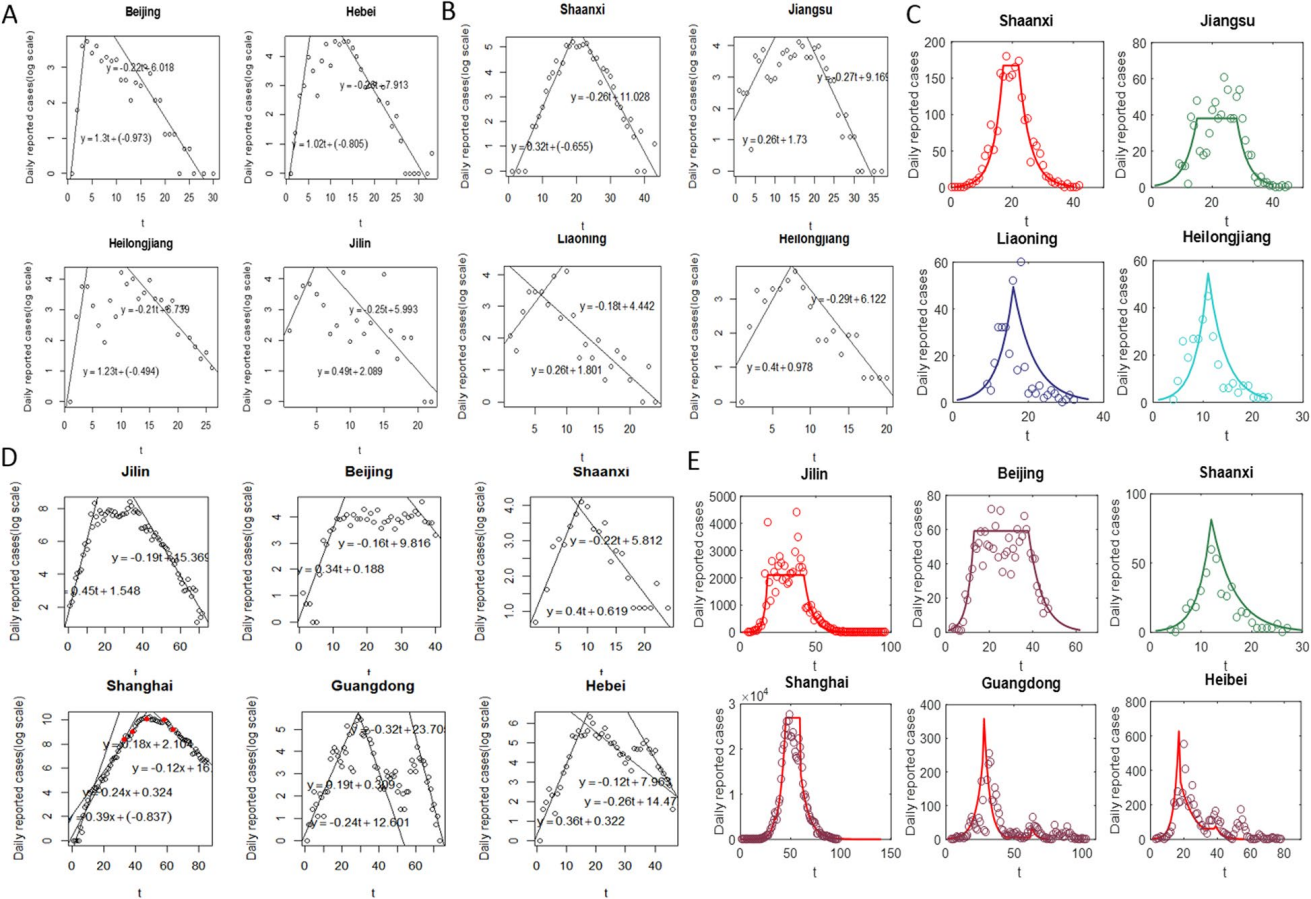
Different stages of epidemic evolutions and the dynamic zeroing processes. Using linear regression lines to fit logarithmic data and growth curves to fit original data caused by the Alpha, Delta and Omicron variants, we analyzed the impact of NPI strategies on dynamic zeroing in four periods. The slope of the rising straight line reflects the severity and risk of the epidemic and the timeliness of the NPIs, and the slope of the falling straight line reflects the strength of the NPI measures. Subplots **a**, **b** and **d** represent the linear regression lines and logarithms of data on epidemics caused by the Alpha, Delta and Omicron variants. Subplots **c** and **e** represent fitting the original data caused by the Delta and Omicron variants, calculated from the linear regression results shown in subplots **b** and **d** (Guangdong and Hebei provinces had slight rebounds during their decline periods, so there are two linear regression fitting curves for these declines)

By taking the COVID-19 epidemics caused by the Alpha strain in Beijing, Hebei, Heilongjiang and Jilin as examples, we carried out a logarithmic analysis of the data in each region by using linear regression equations of different stages in each region to examine the interaction between epidemic evolutions and the dynamic clearing process. During the Alpha epidemic period, the early exponential growth rates were very high (the linear growth slopes of the four regions were 1.3, 1.02, 1.23 and 0.49, respectively; Fig. 3a). Moreover, the exponential growth trends of the four regions were cut off within five days. This shows that China's quarantining of close contacts, rapid nucleic acid screening, locking down of residential communities with active transmissions and imposing travel restrictions can effectively curb epidemics caused by virus strains with long incubation periods and low infectivity in a relatively short time. It also indicates that the speed of implementation of NPIs was faster than the transmission speed of the Alpha strain [3, 25].

 The early linear growth slopes of the Delta mutant causing COVID-19 in Shaanxi, Jiangsu, Liaoning and Heilongjiang Provinces were 0.32, 0.26, 0.26 and 0.4, respectively (Fig. 3b). The epidemic data in Heilongjiang Province grew the fastest in the early stage, but its free rising and containment rising periods were relatively short. In the later stage, the slopes of the linear declines were -0.26, -0.27, -0.18, -0.29, respectively (Fig. 3b-c). The epidemic situation decreased rapidly, and the values of ${R}_{C2}$ (the estimated ${S}_{c}$) were 0.48 (0.84), 0.46 (0.82), 0.64 (0.75) and 0.42 (0.88), respectively (Supplementary materials: Extended Data Table 3). These results show that Heilongjiang had the smallest ${R}_{C2}$, the largest NPI efforts, and consequently the minimum clearing time (Extended Data Table 4). Thus, the strength of NPIs for the Delta virus-induced epidemic was very strong [26], compared with the mean upper limit ${S}_{c}$= 0.89.

In Jilin, Beijing, Shaanxi, Shanghai, Guangdong and Hebei, the early linear growth slopes of the epidemic induced by the Omicron strain were 0.45, 0.34, 0.4, 0.39, 0.19 and 0.36, respectively (Fig. 3d, Supplementary materials: Extended Data Table 2), among which Jilin was the fastest, while Guangdong was the slowest. According to the linear regression fitting results, the free rising period in Shanghai was 0 days, in Beijing and Hebei it was 2 days [27, 28], and in Guangdong and Shaanxi 3 days (Supplementary materials: Extended Data Table 4), indicating that the epidemic situation in most areas was found earlier during Omicron epidemics than in those caused by other variants. Comparing Beijing and Jilin, Jilin was found relatively late, the initial growth rate of Beijing was slightly lower than that of Jilin, and the time to reach the plateau period was 3 days earlier than that for Jilin. Beijing quickly ended the containment period and entered the plateau period, resulting in a huge difference in the development of the epidemic between Beijing and Jilin in the later period (Fig. 3e, Supplementary materials: Extended Data Table 4).

During the exponential decreasing stage, the values of ${R}_{C2}$ (${S}_{c}$) were 0.62 (0.83), 0.68 (0.78), 0.56 (0.83), 0.76(0.77), 0.36 (0.83), 0.48 (0.85) in the above six regions, respectively (Fig. 3d, Supplementary materials: Extended Data Table 3), with Shanghai having the largest ${R}_{C2}$ and the smallest ${S}_{c}$. Note that the slopes of the late linear decline phases of the epidemics induced by the Omicron strain were -0.19, -0.16, -0.22, -0.12, -0.24 (-0.32), -0.16 (-0.26). Two slopes are provided for both Guangdong and Hebei provinces due to slight rebounds that occurred during their decline periods (Fig. 3d). Notice that Shanghai had the slowest decline while Guangdong had the fastest decline. The biggest difference between these two is that Guangdong crossed the plateau period during a short containment of the rise, which rapidly declined. Thus, the scale of this round of the epidemic in Guangdong was far smaller than that in Shanghai, and the clearing time after the decline was significantly faster than that in Shanghai, a difference of 65 days (Supplementary materials: Extended Data Table 4). The linear regression fitting completely reproduces the dynamic adjustment process of NPIs in Shanghai with five time points shown as red dots in Fig. 3d, making the slope of the straight line ($\mathrm{or }{R}_{c1})$ drop from 0.39 (or 3.34) at the earliest to 0.18 (or 2.08) in the rising containment period. It took 44 days to cut off the exponential growth and enter the plateau period with four step-by-step increments in the strength of NPIs, resulting in difficulties in dynamic zeroing in the later period. This emphasizes the importance of quick and intense responses, effectively shortening the exponential growth period.

By comparing values of ${R}_{C2}$ for the epidemics induced by the Delta and Omicron strains we found that, with the same mean ${S}_{c}$, the ${R}_{c2}$ for the epidemic associated with the Delta strain is relatively small, leading to dynamic zeroing being achieved fast [28, 29]. Moreover, most Omicron-induced epidemics were cleared within relatively short times (before 1 June 2022, shown in Fig. 1a, Supplementary materials: Extended Data Table 2), but took far longer to clear than epidemics induced by the Alpha and Delta strains. Therefore, all these results confirm that China's high vaccination rate and strong NPIs can effectively avoid various waves of epidemics induced by existing strains and achieve dynamic zeroing, but there are still great risks and uncertainties with small outbreaks during the declining stage due to prerequisites and threshold levels associated with dynamic zeroing (Fig. 3d-e).

In addition, the epidemic durations (including the plateau phases) for 6 epidemics induced by Omicron and for 4 epidemics induced by Delta strains based on Eq. (9) are shown in Extended Data Table 4, while the real epidemic durations for these 10 epidemics are shown in Extended Data Tables 1 and 2. It should be noted here that when estimating the duration of an epidemic, we consider that the free rising period due to the first reported case for each epidemic inevitably has a certain lag. Thus, the general estimation result is slightly longer than the time series of real data. For example, for the epidemic situation in Liaoning (Heilongjiang) induced by the Delta strain, the estimated epidemic period was 36 days (23 days, Supplementary materials: Extended Data Table 4). If the estimated free rising period of 8 days (3 days) is subtracted, it would have been 28 days (20 days), which is consistent with the reported duration of 24 days (20 days, Supplementary materials: Extended Data Table 1). In addition, the epidemics in Guangdong and Hebei Provinces induced by the Omicron strain showed small fluctuations in the declining stage. In general, the reported durations of the epidemics were 97 and 76 days, respectively (Supplementary materials: Extended Data Table 2), with three peaks (Fig. 3d-e). Specifically, the number of daily reported cases in Guangdong (Hebei) reached 0 on the 75th (47th) day, and then increased when a third small peak appeared. When estimating the duration of an epidemic, we used the exponential decline rate of the second declining stage, namely -0.32 (Guangdong) and -0.26 (Hebei). The estimated durations of the epidemics were 75 days and 56 days, respectively, which were consistent with the actual data.

**Fig. 4:**
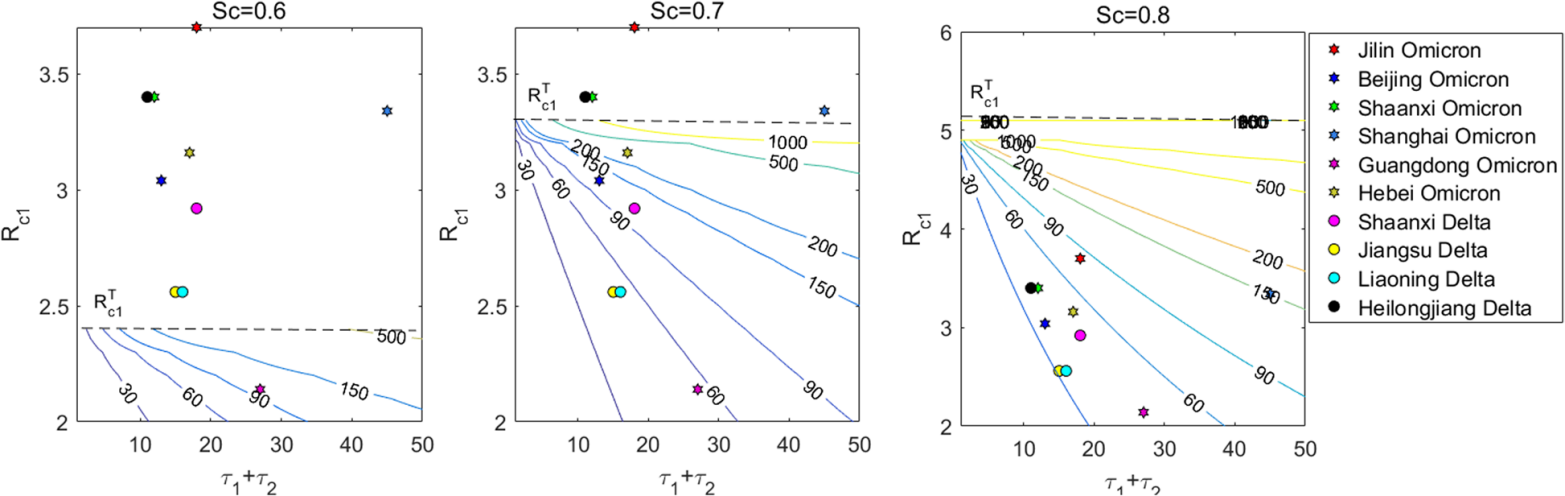
Determination of epidemic duration and threshold level of the NPIs’ strength. Based on the strength of NPIs $({S}_{c})$ and the formula for ${T}_{c}$ without considering the plateau period (${\tau }_{3}$), the maximum value of ${R}_{c1}$ during the exponential growth stage that can be dynamically cleared in the later decline stage under different intensities of NPIs can be obtained. The contour diagram of the clearing time ${T}_{c}$ with respect to the CRN ${R}_{c1}$ during the early EGS, the peak time τ and the intensity of NPI measures ${S}_{c}$. Numbers marked on lines of the figure represent values of ${T}_{c}$, and dots represent the values of ${R}_{c1}$ and τ of these regions. The horizontal line ${R}_{c1}^{T}$ represents the threshold value of whether the epidemic can be dynamically cleared or not, which indicates that the epidemic cannot be cleared once ${R}_{c1}$ exceeds ${R}_{c1}^{T}$

To reveal complex relations between the clearing time ${T}_{c}$ with respect to the ${R}_{c1}$ during the early exponential growth stage (EGS), the peak time τ and the relatively strengthened NPI ${S}_{c}$, we derived the contour plots shown in Fig. 4. These indicate the threshold conditions for clearing or not clearing epidemics induced by the Delta and Omicron strains with different values of ${S}_{c}$. In particular, when ${S}_{c}$= 0.6 (or 0.7, or 0.8), it is impossible to clear epidemics caused by the Delta or Omicron strains once ${R}_{c1}$ exceeds 2.4 (or 3.3, or 5.1). Fortunately, the estimated ${R}_{c1}$ values for all epidemics caused by the Delta and Omicron strains in China were less than these thresholds (Supplementary materials: Extended Data Tables 1 and 2), indicating that the dynamic zero-case policy was being successful.

Moreover, when ${S}_{c}$= 0.7, if the ${R}_{c1}$ reaches 2.5 (or 3) in the EGS, the epidemic could be cleared within one month, provided that the EGS could be cut off within 11 days (5 days). When ${S}_{c}$= 0.8, if the ${R}_{c1}$ reaches 3 in the EGS, the epidemic could be cleared within one month provided that the EGS could be cut off within 12 days, otherwise the epidemic cannot be cleared within two (three) months if the exponential growth trends cannot cut be off within 23 (34) days. In addition, compared with Hebei, the ${R}_{c1}$ in Shaanxi was slightly higher than that in Hebei, but the exponential growth period of Shaanxi was 8 days shorter than that in Hebei, which resulted in the clearing time in Shaanxi being nearly 1 month earlier than that in Hebei. This illustrates that the exponential growth period played an essential role in realizing the dynamic zero-case policy. It is worth noting that the estimated exponential growth periods for Jilin (Shanghai) were 18 days (45 days) (Supplementary materials: Extended Data Tables 3 and 4), so the estimated clearing time of the epidemic in Jilin was nearly 3 months, while the epidemic in Shanghai could be cleared within 3 months, agreeing well with the real scenarios.

In order to compare the epidemic situation without vaccines and control measures with existing vaccine regimes and very strong control measures, we assumed that the basic reproduction number of the Omicron variant was 8 (baseline value), and calculated the cumulative number of infected people within 9 days (${N}_{I}^{9}$) and the percentage decrease in the cumulative number of infected people compared to the baseline value (${P}_{d}$) under different CRNs and the baseline value. As shown in Extended Data Table 5, we found that the total number of infected persons in the nine waves of the Omicron epidemic in China has been reduced by more than 99% compared with its benchmark R_0_. In addition, we also calculated the time required for the number of infected people to reach 10,000. In the case of the Omicron baseline ${\mathrm{R}}_{0}$, it takes 10 days to reach 10,000 infections, while in the nine waves of the Omicron epidemic in China, it takes 41 days when the ${R}_{c1}$ value is the minimum of 2.14 (Guangdong) and 20 days when the ${R}_{c1}$ value is very large (3.7, Jilin). Therefore, China's comprehensive capacity in vaccine and health prevention and control could have delayed the rapid growth of the epidemic for at least 11–32 days, thereby winning some precious time for the comprehensive deployment and strengthening of prevention and control measures.

## Discussion

 Our study found that the real ${R}_{c1}$ values of the Omicron variant in the epidemic of 9 regions in China was only 2.14–3.70 (Supplementary materials: Extended Data Table 2), which was not only far lower than those of South Africa, the United States of America and Hong Kong (6–7.75) [15, 17, 20], but also lower than the value of 3.82 for the original strain in Wuhan. By comparing the real data with simulated infections under the benchmark R_0_ value for Omicron in the 7 regions where the Omicron epidemic had ended as of 1 June 2022, we found that more than 99% of the cases of COVID-19 were prevented (Supplementary materials: Extended Data Table 5). Under the combined effect of vaccines and NPIs, the DZCP maintained the ${R}_{c1}$ of the 101 outbreaks studied in this paper below the safe threshold level, but the strength of NPIs was close to saturation (0.89), and there was little room for improvement. However, the high infectivity and occult nature of mutant strains inevitably leads to difficulties in early detection [30–32], which makes the early exponential growth exceed the threshold for successful dynamic zeroing, leading to increasing uncertainty about the outbreak of new, larger scale, aggregated outbreaks, and even to epidemics becoming out of control due to waning immunity [30–32]. After the “new 10 measures” announced on 7 December 2022 [5, 6], many large cities, including Beijing, Shanghai, Chongqing and Chengdu, have experienced shocking first waves with the number of infected people approaching 70%, which has had huge impacts on the healthcare system. The above results confirm that there exists a critical threshold for the NPI strategy of the DZCP, but when the intensity of NPIs is lower than this threshold, a major outbreak is inevitable. Therefore, further consolidating and strengthening of China's vaccine immune barrier can effectively improve China's ability to prevent and control epidemics and provide greater scope for the selection and adjustment of NPIs.

The models and analysis methods established in this study are not only applicable to the study of small-scale clustering COVID-19 epidemics in China, but are also applicable to the study of epidemic development trends and their durations in other regions or for other diseases. However, due to the assumption that the number of susceptible individuals is approximately equal to the total population, the model can only be applied to small-scale outbreak research caused by a virus with a low transmission rate or with strict control measures, as well as research on the development of epidemic trends during the early stages of an outbreak.

## Conclusions

The main conclusions are that the durations of each outbreak and their dynamic zeroing processes mainly depended on their early exponential growth rates, peak times and peak values, as well as their late exponential decline rates. Moreover, the early or late discovery of each outbreak and the timing when NPIs were started are two key factors that determine the early exponential growth rate and peak value, which naturally affect the duration of each outbreak. The synergistic effect of the above factors affecting the duration of epidemics and the DZCP is an important subject for further in-depth study. Moreover, we did not take into account the impact of factors such as vaccination time and immune waning on the independent effect of the vaccine, which may affect the accuracy of estimates [33, 34]. How to integrate immune dynamic waning and develop more realistic models including age structure and symptomatic and asymptomatic classes is an important aspect for future research [33, 34].

## Supplementary Information

**Additional file 1.**

**Additional file 2.**

## Data Availability

The data used in this study are from the National Health Commission of the People’s Republic of China (http://www.nhc.gov.cn/xcs/xxgzbd/gzbd_index.shtml) and also available in the Supplementary Materials.
