# Supplementary material for: Threshold conditions for curbing COVID-19 with a dynamic zero-case policy derived from 101 outbreaks in China
Source: BMC Public Health. 2023 Jun 6;23:1084. doi: 10.1186/s12889-023-16009-8 (PMC10242611; doi:10.1186/s12889-023-16009-8)
Supplement: Supplementary file 1 — Additional file 1. [file 12889_2023_16009_MOESM1_ESM.pdf]

Supplementary materials 1. Extended data Figure and Tables.

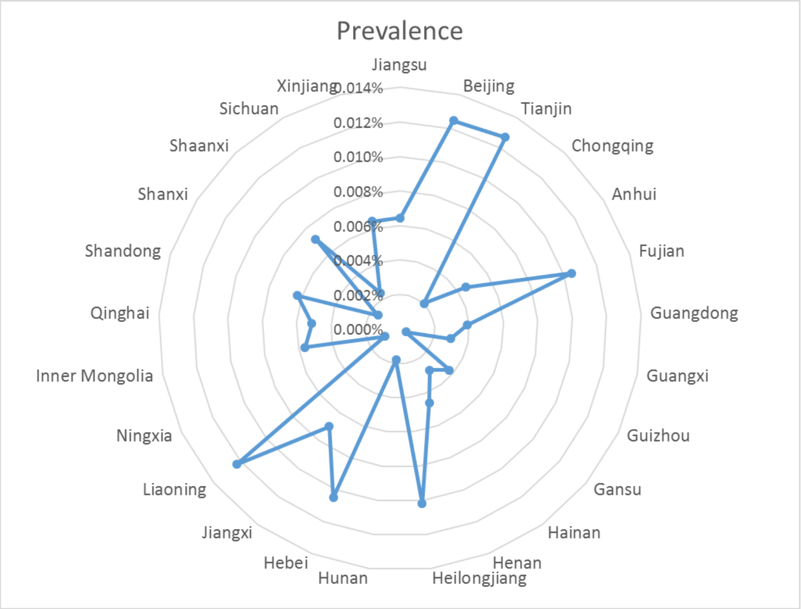

Extended Data Figure 1. Proportions of the total number of infected cases for each outbreak to the total population of each province.

Extended Data Table 1. Basic information on epidemics in some provinces in China induced by the Delta strain in 2021.

Location	Population size	Start time	Total vaccinations	Vaccination ratio	r_1	R_{c1}	Epidemic duration（days）	Peak (days)
Shaanxi	39600000	9 December	68295129	1.73	0.32	2.92	43	180
Liaoning	42600000	4 Nov	69117685	1.62	0.26	2.56	24	60
Inner Mongolia	24000000	13 October	38376425	1.60	0.28	2.68	31	36
Fujian	41900000	10 September	62469089	1.49	0.41	3.46	20	61
Heilongjiang	31300000	27 October	46281031	1.48	0.4	3.40	20	45
Jiangsu (Yangzhou)	85100000	21 July	90728314	1.07	0.26 (0.5)	2.56 (4.0)	37	61

Extended Data Table 2. Basic information on epidemics in some provinces in China induced by the Omicron strain in 2022.

Location	Population size	Starting time	Total vaccinations	Vaccination ratio	r_1	R_{c1}	Epidemic duration （days）	Peak (days)
Beijing	21890000	17 April	58045117	2.65	0.34	3.04	45*	96
Hebei	74600000	2 March	174433264	2.34	0.36	3.16	76	555
Guangdong	127000000	12 February	292078418	2.30	0.19	2.14	97	256
Shanghai	24900000	24 February	55052706	2.21	0.39	3.34	97*	27605
Shandong	102000000	28 February	221923901	2.18	0.42	3.52	69	555
Liaoning	42600000	6 March	84956073	1.99	0.37	3.22	87*	246
Jiangxi	45170000	13 March	88644490	1.96	0.41	3.46	63	137
Shaanxi	39600000	5 March	75676949	1.91	0.4	3.40	24	60
Jilin	24000000	1 March	43919446	1.83	0.45	3.70	92*	4427

*epidemic duration until 31 May.

Extended Data Table 3. The reproduction numbers and prevention and control strangths S_c in the exponential decline stage. These results related to $1/\gamma_2 = 2$ days have been used in the main text.

Location (Omicron)	$\boldsymbol{R_{c1}}$ ($\frac{1}{\gamma_1}$=6)	$\boldsymbol{R_{c1}}$ ($\frac{1}{\gamma_1}$=5)	$\boldsymbol{r_2}$	$\boldsymbol{1/\gamma_2 = 2}$			$\boldsymbol{1/\gamma_2 = 1}$		
				R_{C2}	S_c		R_{C2}	S_c	
					$\frac{1}{\gamma_1}$=6	$\frac{1}{\gamma_1}$=5		$\frac{1}{\gamma_1}$=6	$\frac{1}{\gamma_1}$=5
Jilin	3.70	3.25	-0.19	0.62	0.83	0.81	0.81	0.78	0.75
Beijing	3.04	2.70	-0.16	0.68	0.78	0.75	0.84	0.72	0.69
Shaanxi	3.40	3.00	-0.22	0.56	0.83	0.81	0.78	0.77	0.74
Shanghai	3.34	2.95	-0.12	0.76	0.77	0.74	0.88	0.74	0.70
Guangdong	2.14	1.95	-0.32	0.36	0.83	0.82	0.68	0.68	0.65
Hebei	3.16	2.80	-0.26	0.48	0.85	0.83	0.74	0.77	0.74
Mean	3.13	2.78	-0.21	0.58	0.81	0.79	0.79	0.74	0.71
Location (Delta)	R_{c1} ($\frac{1}{\gamma_1}$=**6**)	R_{c1} ($\frac{1}{\gamma_1}$=**5**)	$\boldsymbol{r_2}$	$1/\gamma_2 = 2$			$1/\gamma_2 = 1$		
				R_{C2}	S_c		R_{C2}	S_c	
					$\frac{1}{\gamma_1}$=6	$\frac{1}{\gamma_1}$=5		$\frac{1}{\gamma_1}$=6	$\frac{1}{\gamma_1}$=5
Shaanxi	2.92	2.60	-0.26	0.48	0.84	0.82	0.74	0.75	0.72
Jiangsu	2.56	2.30	-0.27	0.46	0.82	0.80	0.73	0.71	0.68
Liaoning	2.56	2.30	-0.18	0.64	0.75	0.72	0.82	0.68	0.64
Heilongjiang	3.40	3.0	-0.29	0.42	0.88	0.86	0.71	0.79	0.76
Mean	2.86	2.55	-0.25	0.51	0.82	0.80	0.75	0.73	0.70

Extended Data Table 4. Estimated durations of the four stages of COVID-19 for the epidemics in some provinces in China induced by the Omicron and Delta strains, respectively.

Location (Omicron)	Free rising ($\boldsymbol{\tau_1}$)	Containment ($\boldsymbol{\tau_2}$)	Plateau ($\boldsymbol{\tau_3}$)	Exponential decline ($\boldsymbol{\tau_4}$)	Epidemic duration ($\boldsymbol{T_c}$)
Jilin	4	14	24	39	81
Beijing	2	11	25	21	59
Shaanxi	3	9	0	18	30
Shanghai	0	45	14	81	140
Guangdong	3	24	0	48	75
Hebei	2	15	0	39	56
**Location (Delta)**	**Free rising (τ_1)**	**Containment (τ_2)**	**Plateau (τ_3)**	**Exponential decline (τ_4)**	**Epidemic duration (T_c)**
Shaanxi	0	18	5	18	40
Jiangsu	8	7	13	12	40
Liaoning	8	8	0	20	36
Heilongjiang	3	8	0	12	23

Extended Data Table 5. Comparison of Omicron epidemic development under different reproduction numbers with the theoretical value calculated with the basic reproduction number = 8, when $\gamma_1 = 1/6$.

Location	R_{c1}	N_I^9	P_d	T	Delay (days)
baseline	8	16422	-	9	-
Beijing	3.04	50	99.69	25	16
Hebei	3.16	57	99.66	24	15
Guangdong	2.14	22	99.87	41	32
Shanghai	3.34	68	99.59	22	13
Shandong	3.52	82	99.50	21	12
Liaoning	3.22	60	99.63	23	14
Jiangxi	3.46	77	99.53	21	12
Shaanxi	3.4	72	99.56	22	13
Jilin	3.70	99	99.40	20	11

N_I^9: Cumulative number of infected persons in 9 days.

P_d : Percentage reduction in the number of infected persons.

T: Time required for the number of infected people to reach 10000.

Delay: Time difference between number of days (10) for the cumulative number of cases to reach 10000 with a basic reproduction number of 8 and when the CRN value is modelled.

Extended Data Table 6. The contacts c and the relatively strengthened NPIs S_c under different circumstances.

c_1/c_0	c_1	$c_2/c_1 = 2/3$		$c_2/c_1 = 1/2$		$c_2/c_1 = 1/3$	
		c	S_c	c	S_c	c	S_c
2/3	9.3/13.3	6.2/8.7	0.78	4.7/6.7	0.83	3.1/4.4	0.89
1/2	7/10	4.7/6.7	0.78	3.5/5	0.83	**2.3/3.3**	0.89
1/3	4.7/6.7	3.1/4.5	0.78	**1.6/2.3**	0.83	**1.6/2.2**	0.89

Extended Data Table 7. Sensitivity analyses of the CRNs and the relatively strengthened NPIs S_c in the exponential decline stage for different $1/\gamma_2$ (here $\gamma_1 = 1/7$).

Location (Omicron)	R_{c1} ($\gamma_1 = 1/7$)	r_2	$1/\gamma_2 = 2$		$1/\gamma_2 = 1$	
			R_{C2}	S_c	R_{C2}	S_c
Jilin	4.15	-0.19	0.62	0.85	0.81	0.80
Beijing	3.38	-0.16	0.68	0.80	0.84	0.75
Shaanxi	3.8	-0.20	0.6	0.84	0.8	0.79
Shanghai	3.73	-0.12	0.76	0.80	0.88	0.76
Guangdong	2.33	-0.32	0.36	0.85	0.68	0.71
Hebei	3.52	-0.26	0.48	0.86	0.74	0.79
Mean	3.49	-0.21	0.58	0.83	0.79	0.77
Location (Delta)	R_{c1} ($\gamma_1 = 1/7$)	r_2	$1/\gamma_2 = 2$		$1/\gamma_2 = 1$	
			R_{C2}	S_c	R_{C2}	S_c
Shaanxi	3.24	-0.26	0.48	0.85	0.4	0.77
Jiangsu	2.82	-0.27	0.46	0.84	0.73	0.74
Liaoning	2.82	-0.18	0.64	0.77	0.82	0.71
Heilongjiang	3.8	-0.29	0.42	0.89	0.71	0.81
Mean	3.17	-0.25	0.51	0.84	0.75	0.76

Supplementary materials 2. Method of the estimation of change points

We used the linear regression equation model with the control reproduction number switching to fit the logarithm of the data linear regression equations. Each switching point is individually estimated by a Bayesian approach. The Bayes' linear regression model is

$$Y = B\beta + \varepsilon, \varepsilon \sim N(0, \sigma^2).$$

The posterior distribution of a single change point for a general switching linear model as described by Chin and Broemeling (1980). For a series of discrete time data, the possible value of a single change point in the series is the corresponding time for each data point. We assume that the prior distribution of each change point obeys an uniform distribution with the length of the data. The estimated value of the changing point is generated from the posterior distribution based on the prior information. The specific steps are as follows.

Step 1 Give the prior distribution of the change point based on the time range within which switching points may occur:

$$p(T) = U(T - M, T + M)$$

Step 2 Give the prior parameters values (n_0, s_0, m, V) of the Bayes linear model. The prior distribution of $1/\sigma^2$ is the gamma distribution, n_0 and s_0 are prior parameters. m and V are the prior mean vector and variance matrix of β, respectively.

Step 3 Generate the sample of change points on the time interval [T−M, T+M] from the posterior distribution,

$$\mathrm{P}(\hat{T}|\mathrm{Data}) \propto D(\hat{T})^{-n_1} |Q_1|^{-1/2}$$

where

$$n_1 = n_0 + \frac{N}{2};$$

$$D(\hat{T}) = s_0 + \frac{[(Y - B\beta_1)'Y + (m - \beta_1)'Q_0 m]}{2};$$

$$\beta_1 = Q_1^{-1}[Q_0 m + B'Y];$$

$$Q_1 = B'B + Q_0;$$

$$Q_0 = V^{-1}\frac{s_0}{n_0}.$$

the maximum value of the distribution is the optimum change point. Each change point is obtained in the same way.
